# Effect of Spark Plasma Sintering Parameters and Thermomechanical Processing on the Microstructural Evolution of the Ti-10V-2Fe-3Al Titanium Alloy

**DOI:** 10.3390/ma19214738

**Published:** 2026-11-08

**Authors:** Marcin Knapiński, Teresa Bajor, Grzegorz Boczkal, Małgorzata Perek-Nowak

**Affiliations:** 1 Faculty of Production Engineering and Materials Technology, Czestochowa University of Technology, 19 Armii Krajowej, 42-201 Czestochowa, Poland; marcin.knapinski@pcz.pl; 2 Faculty of Non-Ferrous Metals, AGH University of Krakow, 30 Mickiewicza Ave, 30-059 Krakow, Polandmperek@agh.edu.pl (M.P.-N.)

**Keywords:** Ti-10V-2Fe-3Al titanium alloy, spark plasma sintering (SPS), powder metallurgy, hot plastic deformation, microstructural evolution, phase transformations

## Abstract

This paper presents the effect of spark plasma sintering (SPS) parameters and hot plastic deformation on the microstructural evolution of the Ti-10V-2Fe-3Al titanium alloy. The study examines the effects of sintering temperature (1200 °C and 1300 °C) and sintering time (10 min and 15 min), as well as plastic deformation parameters, on microstructural transformations. It was demonstrated that the short duration of high-temperature exposure during SPS sintering favours the preservation of local differences in chemical composition, leading to the formation of regions with varying vanadium and other alloying element contents, as well as varying microhardness. Hot deformation tests at 680 °C revealed a stress peak, followed by softening, indicating that structural reorganisation occurs during deformation. It was also found that the intensity of plastic deformation and the sintering temperature influence the homogenization of the material’s chemical composition throughout its volume, the grain refinement of the β phase, and the change in the morphology of the α phase. The use of a two-stage hot plastic deformation process enabled the formation of a fine-grained, banded α + β two-phase structure in a sintered part produced at 1300 °C. The results indicate that the appropriate selection of sintering parameters and subsequent stages of plastic deformation are key tools for shaping the microstructure of the Ti-10V-2Fe-3Al alloy, thereby enabling control over its chemical and structural heterogeneity.

## 1. Introduction

Metastable β-type titanium alloys constitute an important group of structural materials due to their high specific strength and fracture resistance. Compared with structural steels, titanium alloys are characterised by a favourable strength-to-density ratio; therefore, they are used in structures where it is essential to achieve the required mechanical properties while minimising component weight [1,2,3]. Due to their high strength, metastable β titanium alloys are used in the aerospace industry, including for the manufacture of structural components subjected to significant loads [4,5].

Among metastable β-titanium alloys, the Ti-10V-2Fe-3Al alloy, also referred to as Ti-10-2-3 or Ti-1023, is of particular importance. The Ti-10V-2Fe-3Al alloy contains 10% by weight V, 2% by weight Fe, and 3% by weight of Al. Vanadium and iron are β-phase-stabilizing elements, while aluminium stabilises the α-phase. This selection of alloying elements allows the formation of a metastable β matrix after appropriate heat treatment and enables the material to be strengthened by ageing through the precipitation of secondary phases [6,7,8].

An important feature of Ti-10V-2Fe-3Al is the ability to modify its microstructure through the appropriate selection of supersaturation, cooling, and ageing conditions [9,10]. The results of the studies described in [11,12] indicate that the appropriate selection of heat treatment parameters is a fundamental means of shaping the microstructure and mechanical properties of Ti-10V-2Fe-3Al.

The conditions of plastic deformation and subsequent heat treatment significantly affect the microstructure of Ti-10V-2Fe-3Al. In [13], it was found that the method of plastic deformation affects the morphology of the α phase and, consequently, the alloy’s ductility and fracture resistance. A change in the morphology of the primary α phase can lead to different material behaviour during deformation and fracture. Other studies have confirmed the importance of oversaturation and ageing conditions for achieving a favourable combination of mechanical properties, including strength, ductility, fracture resistance, and fatigue properties [14].

A significant problem associated with conventional methods of producing Ti-10V-2Fe-3Al is the potential for local inhomogeneities in the microstructure and chemical composition. The formation of inhomogeneities may be related to the segregation of alloying elements, particularly iron, during the solidification of the alloy. Areas with altered chemical composition may be characterised by a different proportion of the α phase and its morphology. It has been shown that such inhomogeneities can adversely affect the ductility and fatigue life of the material and promote crack initiation [15,16].

Conventional Ti-10V-2Fe-3Al manufacturing technology primarily involves melting and casting the material, followed by plastic deformation—most often through forging—and appropriate heat treatment. The authors of [17] demonstrated that achieving the desired mechanical properties of Ti-10V-2Fe-3Al forgings—including adequate strength, ductility, and fracture resistance—depends, among other factors, on the chemical homogeneity of the starting material, forging parameters, heat treatment conditions, and the thickness of the forging’s cross-section. At the same time, conventional technology can result in significant material loss during subsequent processing and machining stages. In the case of titanium alloys, this issue is particularly significant due to the high cost of the material and its processing [18].

For this reason, alternative manufacturing technologies based on powder metallurgy are being developed; an unquestionable advantage of this approach is the ability to reduce material losses, minimise the number of processing steps, and obtain a material with a more homogeneous chemical composition. Reference [18] presents the possibility of manufacturing components from the Ti-10V-2Fe-3Al alloy using powder metallurgy methods, employing both alloy powder and powder mixtures.

In [19], the course of material densification during sintering and the effect of titanium powder particle size on the resulting density were analysed. The study utilised a mixture of titanium powder and alloying-element powder. It was demonstrated that during sintering, the material undergoes gradual densification without forming a liquid phase, and the resulting density depends largely on the particle size of the titanium powder used. At the same time, it was found that the key challenge is not merely achieving high density, but rather obtaining a homogeneous microstructure and uniform distribution of alloying elements, particularly vanadium [20]. These results indicate that powder metallurgy may be an interesting alternative to conventional methods for producing the Ti-10V-2Fe-3Al alloy; however, the manner in which the consolidation process is conducted significantly affects the structure and properties of the final material.

The research described in [19] confirms that material produced using powder metallurgy methods can undergo further plastic deformation; however, its properties depend on both the consolidation conditions and the parameters of subsequent processing.

An alternative to conventional sintering is a method that utilises the simultaneous application of temperature and pressure, including Spark Plasma Sintering (SPS). This process enables rapid consolidation of powders using an electric current and uniaxial pressure. The most important features of SPS include a high heating rate, a short holding time, and the ability to achieve a density comparable to that obtained in the casting process, while limiting the duration of high-temperature exposure to the material [21,22]. In the case of titanium alloys, SPS process parameters can influence the degree of densification, grain size, and final microstructure [23,24].

Based on a review of the literature, it can be concluded that the SPS method is used for the consolidation of various titanium alloys, and that its parameters have a significant influence on the resulting density and microstructure of the material. In the case of the Ti-10V-2Fe-3Al alloy, a key issue remains determining the influence of SPS process parameters on the structure and properties of the resulting material. As shown above, in the available international literature on the titanium alloy selected for this study, there are many publications concerning the homogenisation of chemical composition and the influence of microstructure, which is shaped by plastic deformation or heat treatment. However, the studies described concern material that is traditionally cast or hot-pressed over several hours. By contrast, there are no publications analysing the influence of SPS process parameters, as well as subsequent thermomechanical treatment of the alloy produced by the SPS process, on its microstructure and, consequently, its performance properties. The authors anticipated that, in the SPS process, the short sintering time of the powders comprising the elemental constituents of an alloy with a high vanadium content would hinder, or indeed prevent, full homogenisation of the chemical composition; they therefore adopted a broader research plan that also included an analysis of the effect of thermomechanical treatment on the microstructure of the Ti-10V-2Fe-3Al. For this reason, the study investigated the consolidation of the alloy using the SPS method, as well as the effect of thermomechanical processing on its microstructure.

The aim of this study is to assess the feasibility of consolidating elemental powders to produce a Ti-10V-2Fe-3Al alloy using the Spark Plasma Sintering (SPS) method, followed by further thermomechanical treatment. The scope of the study includes determining the effects of sintering parameters, hot plastic deformation, subsequent heat treatment, and thermoplastic processing on the density, microstructure, and selected properties of the resulting material. The research also shows that, despite the alloy’s initially highly heterogeneous microstructure produced by the SPS method, the application of appropriate thermoplastic processing can yield a material with an interesting composite microstructure and potentially very promising performance properties.

## 2. Materials, Equipment and Research Methodology

**Figure 1:**
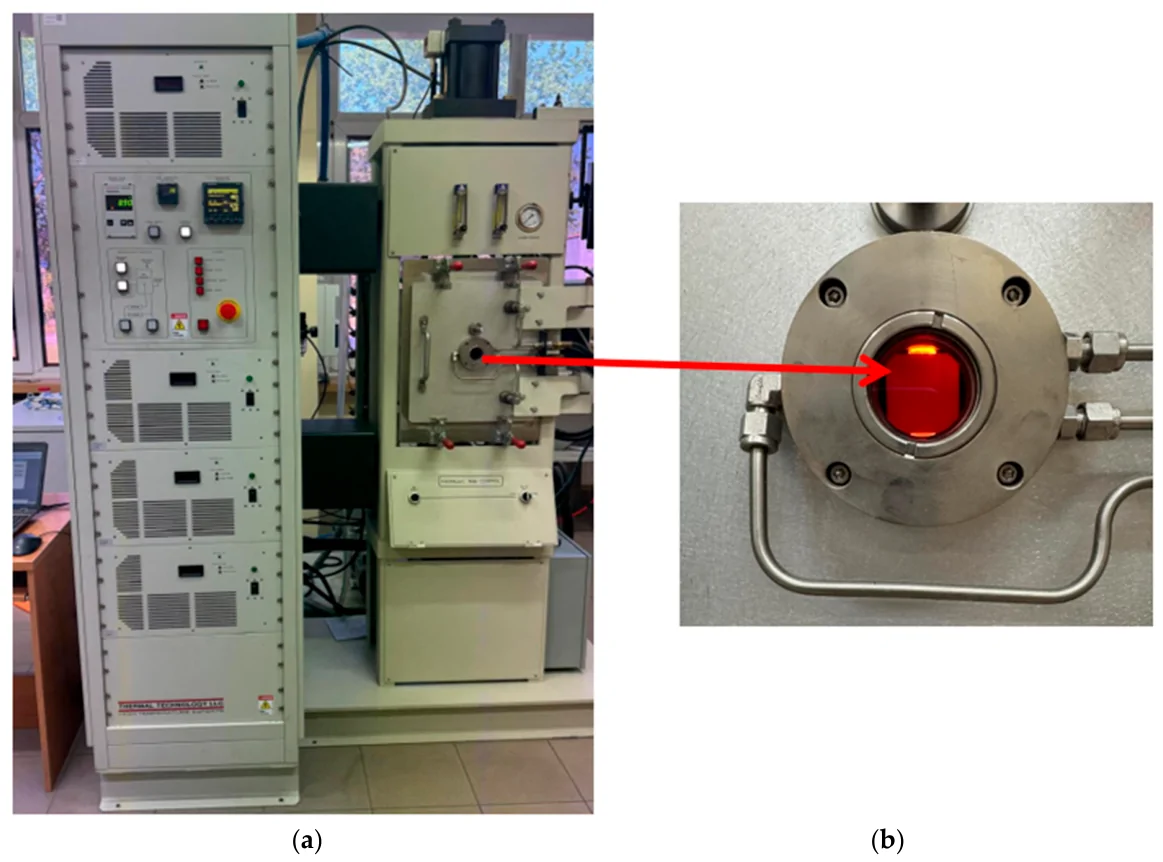
View of the 10-4 spark plasma sintering (SPS) device (**a**) and the sample setup during the process (**b**).

A mixture of elemental components, whose chemical composition is presented in Table 1, was used to produce a Ti-10V-2Fe-3Al titanium alloy sinter. In the first stage of material preparation, the powder was mixed in a Turbula T2F mixer (Tomasova Lea S.R.O., Pardubice, Czech Republic). The mixing time was approximately 1 h. The powder prepared in this manner was sintered using the spark plasma sintering (SPS) method [25,26]. The process was carried out using an SPS 10-4 device from Thermal Technology LLC (Minden, NV, USA) (Figure 1), located at the Faculty of Production Engineering and Materials Technology at the Czestochowa University of Technology. Sintering was performed under vacuum at specified process pressure and temperature. Graphite tooling made of Mersen grade 2333 graphite (Mersen, Paris, France) was used to carry out the sintering process. The prepared powder was placed in the working chamber of the graphite die. Mersen Papyex N998 graphite foil (Mersen, Paris, France) was placed between the powder and the surfaces of the die and punches. The use of the foil facilitated removal of the sample from the die after sintering was complete. The assembly prepared in this manner was placed in the working chamber of the SPS device.

The sintered specimens measured 10 mm in diameter and 15 mm in height, while the SPS process variants differed in their sintering parameters (Table 2). A pressing pressure of 50 MPa and a constant heating rate of 100 °C/min were applied. The pulse duration was 15 ms, and the pause time between pulses was 20% of the pulse duration. The vacuum in the chamber during the process was 5 Pa. The cooling rate after sintering was up to 100 °C/min. The main process parameters, including sintering temperature and holding time, are presented in Table 2. After the sintering process was completed and the setup had cooled, the resulting specimens were removed from the die and then subjected to further testing.

The density of the resulting sintered bodies was determined using the Archimedes method in accordance with the PN-EN ISO 2738:2026-07 standard [27]. Before measurement, the samples were dried, and their mass in air was then determined. In the next step, the samples were saturated with demineralised water, after which their mass was measured both in air and while submerged in water. The measurements were performed using an Ohaus analytical balance (Parsippany, NJ, USA) equipped with a kit for determining solid density, and the resulting values are presented in Table 3.

The density of the samples was determined based on the following Equation (1):

(1) $$\rho =\frac{m_{1}\times \rho_{w}}{m_{3}-m_{4}}$$

where

*m*_1_—dry sample mass;

*m*_3_—mass of the sample weighed above water after soaking;

*m*_4_—mass of the sample weighed in water after soaking;

*ρ_w_*—water density, dependent on ambient temperature.

Microscopic observations of the structures after the sintering process were performed using a Nikon optical microscope (Nikon, Tokyo, Japan), a Phenom XL scanning electron microscope (SEM), and a Hitachi S-3400N scanning electron microscope (Hitachi, Tokyo, Japan) equipped with a tungsten thermionic emission electrode and a special EDS analysis attachment.

Metallographic examinations using light microscopy were carried out on appropriately prepared sample cross-sections (the observation area covered the surface perpendicular to the punch’s axis of compression). The samples were embedded, ground using sandpaper of various grits, polished, and then etched in a two-stage process: in the first stage with 4% HF + 96% H_2_O, and in the second with 2% HF + 2% HNO_3_ + 96% H_2_O.

Hardness measurements were performed using the Vickers method. An FM-700 microhardness tester (Future-Tech Corp., Kawasaki City, Kanagawa Prefecture, Japan) was used for the tests. Studies on the effect of plastic deformation on the structure of materials obtained through sintering were conducted using the Gleeble 3800 metallurgical process simulator with the Hydrawedge II module (DSI Inc., Poestenkill, NY, USA), installed at Czestochowa University of Technology. The tests involved isothermal swelling of cylindrical specimens with a diameter of 10 mm and a height of 13 mm. The specimens were obtained by grinding the cylindrical bases of green compacts produced by the SPS method. Based on a literature review, particularly the results reported in [20,24,25], the authors expected that the SPS sintering process would not yield a material with a microstructure similar to that of a casting alloy. Therefore, they sought to determine whether applying plastic deformation at temperatures below 1000 °C could significantly improve the composite microstructure obtained in the SPS process. The analysis examined the effect of continuous plastic deformation with a true strain (e) of 0.5 applied at a rate of 0.003 s^−1^ and a true strain (ε) of 1.0 applied at a rate of 0.006 s^−1^ at a temperature of 680 °C, i.e., the two-phase range in which the phase α + β occurs in titanium. Two different strain rates were used to maintain a constant strain time. The alloy under study exhibits very good plastic properties in this temperature range during deformation at low strain rates. In addition, the deformation process of die-castings was analysed under two deformation conditions performed at different temperatures. Both deformations had a true strain (ε) of 0.5, applied at a constant rate of 0.003 s^−1^. The first deformation was performed at 980 °C, after which the material was cooled at 2 °C/s to 680 °C, and the second deformation was then applied. The aforementioned optical and scanning microscopes were used to observe the microstructure of the material after plastic deformation.

## 3. Results and Discussion

### 3.1. Analysis of the Powder Components

**Figure 2:**
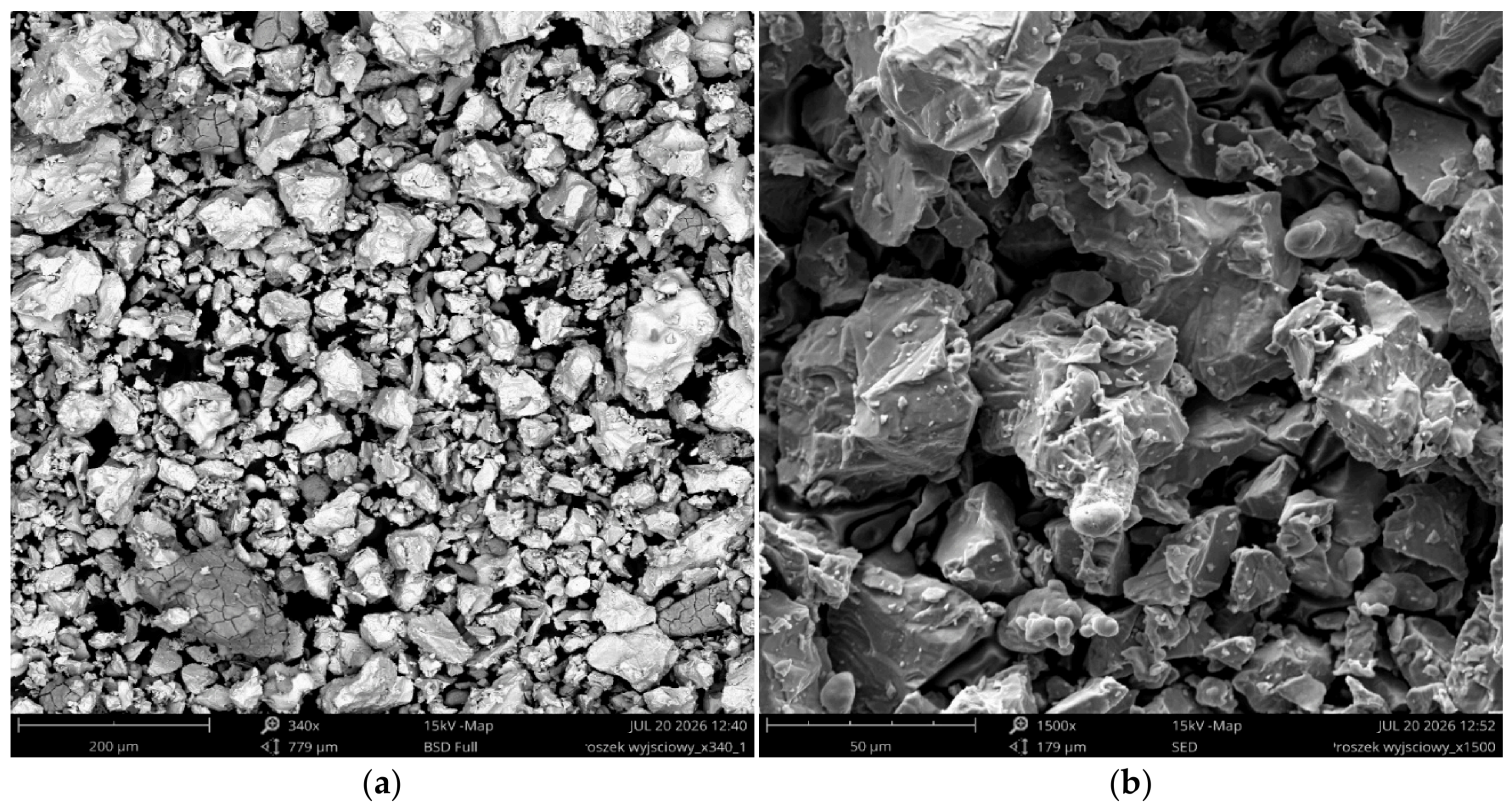
Morphology of the powder mixture of the alloy under investigation, shown in SE images at a magnification of (**a**) 340× and (**b**) 1500×.

**Figure 3:**
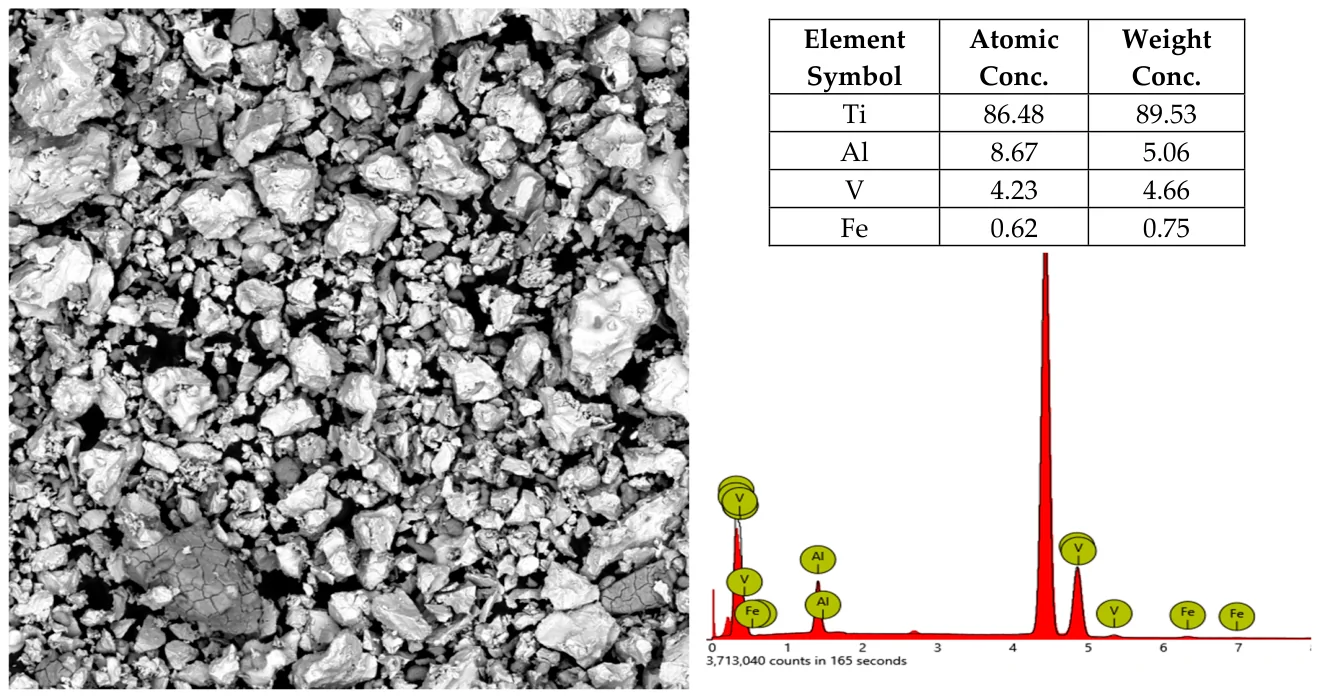

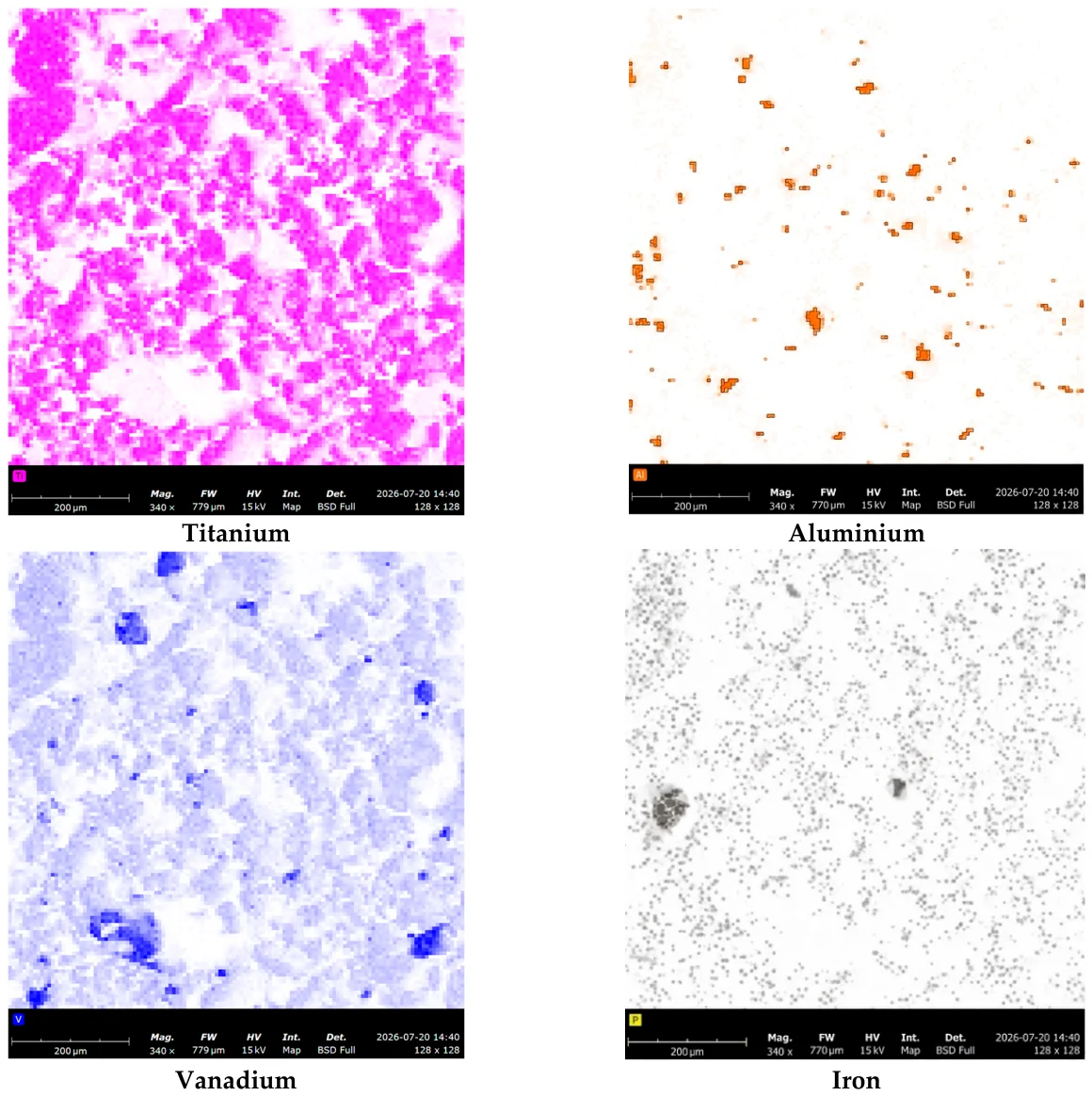
Analysis of the composition of the test alloy powder mixture.

Prior to sintering, the powders were examined using a Phenom XL scanning electron microscope (SEM). The mixture used to prepare the composite sintered body was analysed. The powders were imaged at magnifications ranging from 340× to 1500× in SE contrast (Figure 2 and Figure 3).

The mixture of metal powders exhibits various properties. Depending on the chemical element, particles take on different forms: polyhedral and irregular particles with sharp edges and high density. Titanium powder with a particle size of <150 mm, vanadium powder with a particle size of <100 μm, and aluminium and iron powders with particle sizes of <45 μm were used to produce the sintered bodies. In some areas, irregularly shaped particles with a darker colour can be observed. To identify the chemical composition of individual fractions, an EDS analysis was conducted, the results of which are presented in Figure 3.

### 3.2. Analysis of the Microstructure of SPS-Produced Compacts

**Figure 4:**
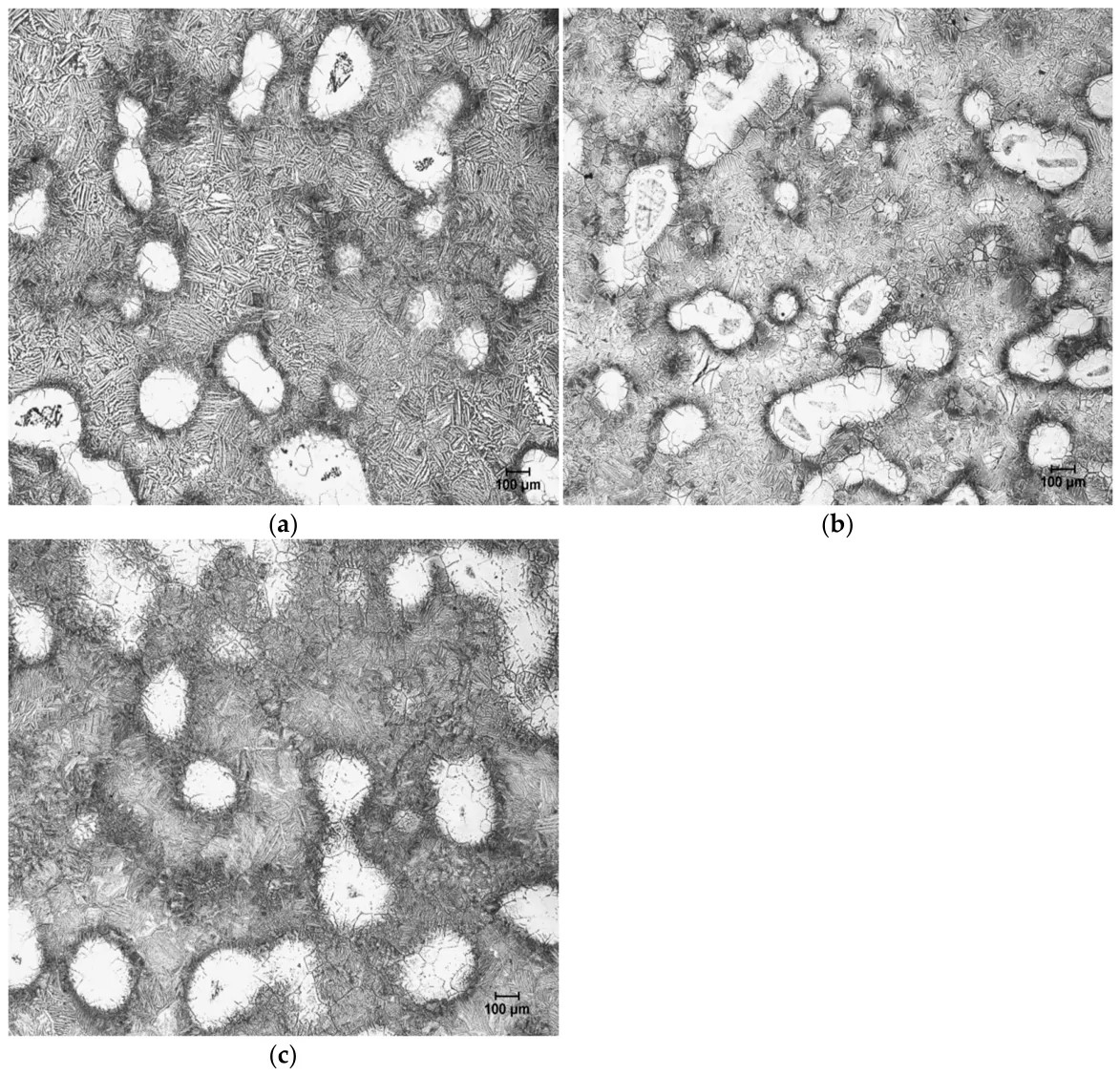
Microstructure of Ti-10-2-3 sintered bodies obtained by the SPS method under the following pressing conditions: Variant I (**a**), Variant II (**b**), and Variant III (**c**).

Figure 4 shows microstructure images obtained during observation of the compacts using an optical microscope. The material was compacted for 10 min at 1200 °C—Variant I (Figure 4a), 15 min at 1200 °C—Variant II (Figure 4b), and 10 min at 1300 °C—Variant III (Figure 4c).

SPS sintering resulted in a highly heterogeneous, composite material. This structure was formed due to the short sintering time characteristic of SPS processes. Despite the very high density of the sintered part, vanadium particles remained in its structure, as they did not fully diffuse into the titanium matrix in such a short time. By examining the structures shown in Figure 4a–c, various regions can be identified: large bright areas with a darker centre represent titanium grains in the β phase, heavily enriched with vanadium. Within these areas, undissolved vanadium particles are visible. These areas are located within the alloy matrix characteristic of the alloy under study, which is a mixture of α and β titanium phases. This structure consists of alternately arranged plates of the α and β phases, formed within the former titanium grains in the β phase that were present at high sintering temperatures.

A comparison of the microstructures obtained in three different sintering conditions reveals that in the first condition (Variant I), the matrix is characterized by the largest, massive precipitates of the α phase of titanium (Figure 4a). As the sintering time was extended or the temperature increased (Figure 4b,c), a slight decrease in the size of the α-phase plates precipitating in the material was observed. At the same time, an increase in sintering temperature caused significant grain growth of the β phase, within which a plate-like α phase structure precipitates during cooling. This phenomenon is particularly evident when comparing the two-phase regions in Figure 4a,c. Another distinguishing feature of the structures is the distinct blurring of the boundary between the large bright regions and the matrix. This can be explained by the differing diffusion kinetics of vanadium in titanium at temperatures of 1200 °C and 1300 °C. The images (Figure 4a) therefore show a diffusion front in the form of darker halos surrounding large, bright titanium regions that are heavily enriched with vanadium.

**Figure 5:**
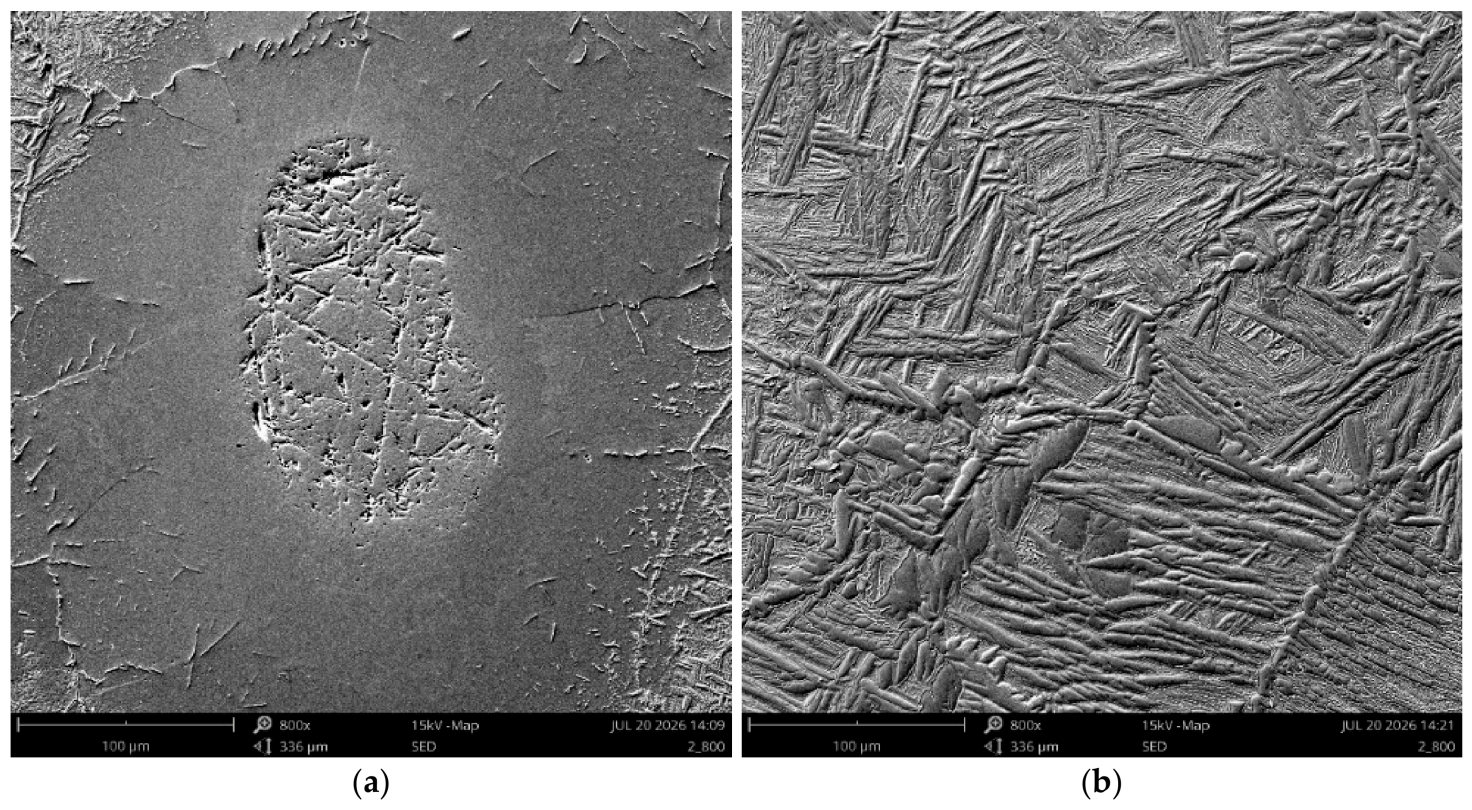
Microstructural image at 800× magnification, showing a remnant vanadium particle (**a**), lamellar structure (α + β) (**b**).

Observations of the microstructure using an optical microscope were supplemented by analysis using scanning electron microscopy (SEM). Figure 5a shows an example of the microstructure of the material for the (1200 °C, 15 min) Variant II. In the central part of the image, an undissolved vanadium particle is visible, surrounded by β-phase grains with an elevated vanadium content. In this region, no precipitation of the α phase is observed; it appears only at a certain distance from the vanadium particle. The α phase initially precipitates along the grain boundaries of the β phase and subsequently also within the former grains of this β phase, forming a lamellar structure (Figure 5b).

**Figure 6:**
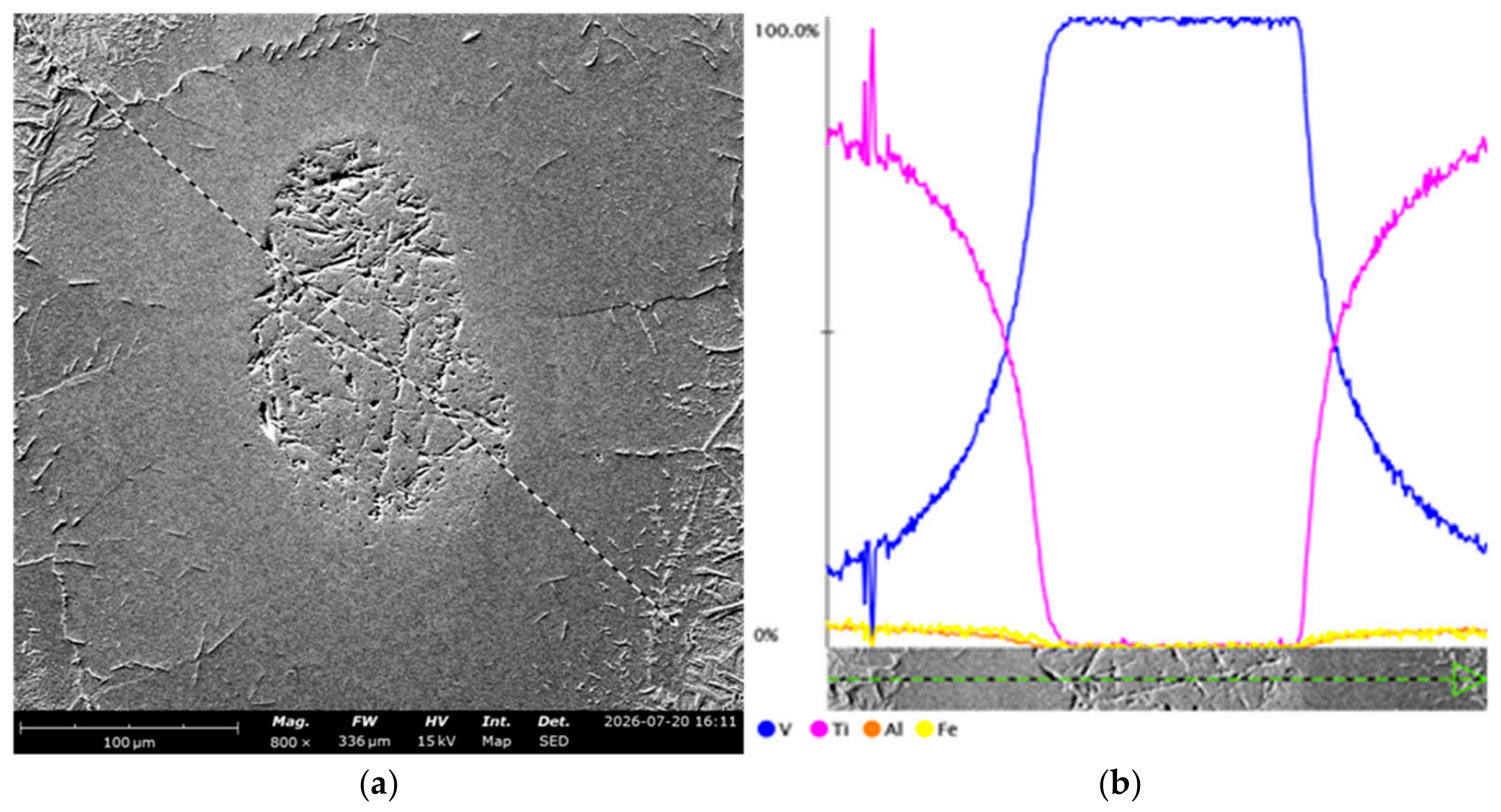
Linear analysis of the chemical composition of the sintered body obtained according to Variant II in the region of the undissolved vanadium particle; SEM-SED image (**a**), detection of the content of Ti, V, Fe, and Al (**b**).

The microstructural observations were supplemented by a linear analysis of the elemental concentration distribution in the process. Figure 6a shows the area of the sample containing an undissolved vanadium particle, along with a line drawn along it, along which the concentration distribution of the elements Ti, V, Fe, and Al, as graphically illustrated in Figure 6b. This analysis confirms earlier observations and explains the morphology of the resulting sintered body.

**Figure 7:**
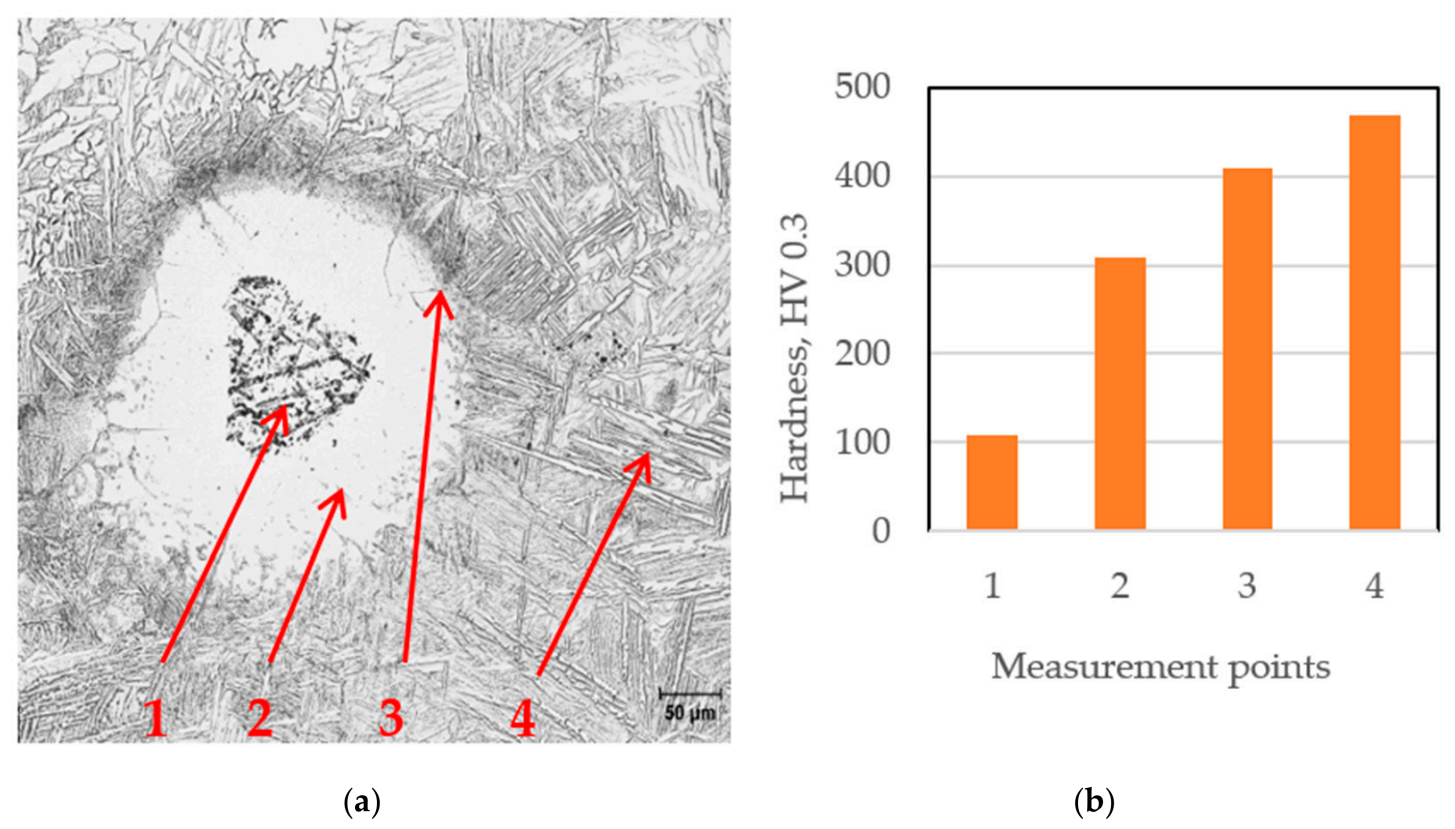
Microhardness test results for the sintered material produced according to Variant II in the area of the undissolved vanadium particle; microstructure image (**a**), hardness measurement results obtained at the points marked in the image (**b**).

The next stage of the research involved Vickers microhardness measurements at a load of 0.3 kgf (PN-EN ISO 6507-1), performed on a casting produced using the SPS process (Variant II). The results are shown in Figure 7b.

The first measurement point was the vanadium phase (Figure 7a). The measured hardness value of this structural component was 109 HV 0.3. For comparison, the hardness of pure, annealed vanadium is approximately 85 HV [28]. The slightly higher value measured in the investigated material may be attributed to microstructural changes that occur during SPS sintering under pressure. These changes may involve microstructural rearrangement and densification, as well as local variations in chemical composition.

The next point of analysis was the area marked with the number 2 in Figure 7a. The microhardness of this phase was 309 HV0.3, confirming that it contains a vanadium-doped titanium β phase. At point 3, at the boundary of the two-phase region, the measured microhardness was 409 HV0.3. The area marked with the number 4 in Figure 7a constituted the largest portion of the analysed structure. The high hardness (470 HV0.3) of the SPS sinter obtained at 1200 °C is likely due to chemical segregation. This region is depleted in vanadium, which did not diffuse completely throughout the material, but is strongly enriched in aluminium, which, as an element that promotes the formation of the α phase, may increase its hardness. At the same time, one must consider the possibility that other factors influence the hardness values obtained, such as microstructural changes during sintering, the degree of material compaction, and potential interactions at interphase boundaries. These factors may affect the local mechanical properties of the tested material and, consequently, the results of microhardness measurements.

### 3.3. Results of the Study on the Effect of Plastic Deformation

**Figure 8:**
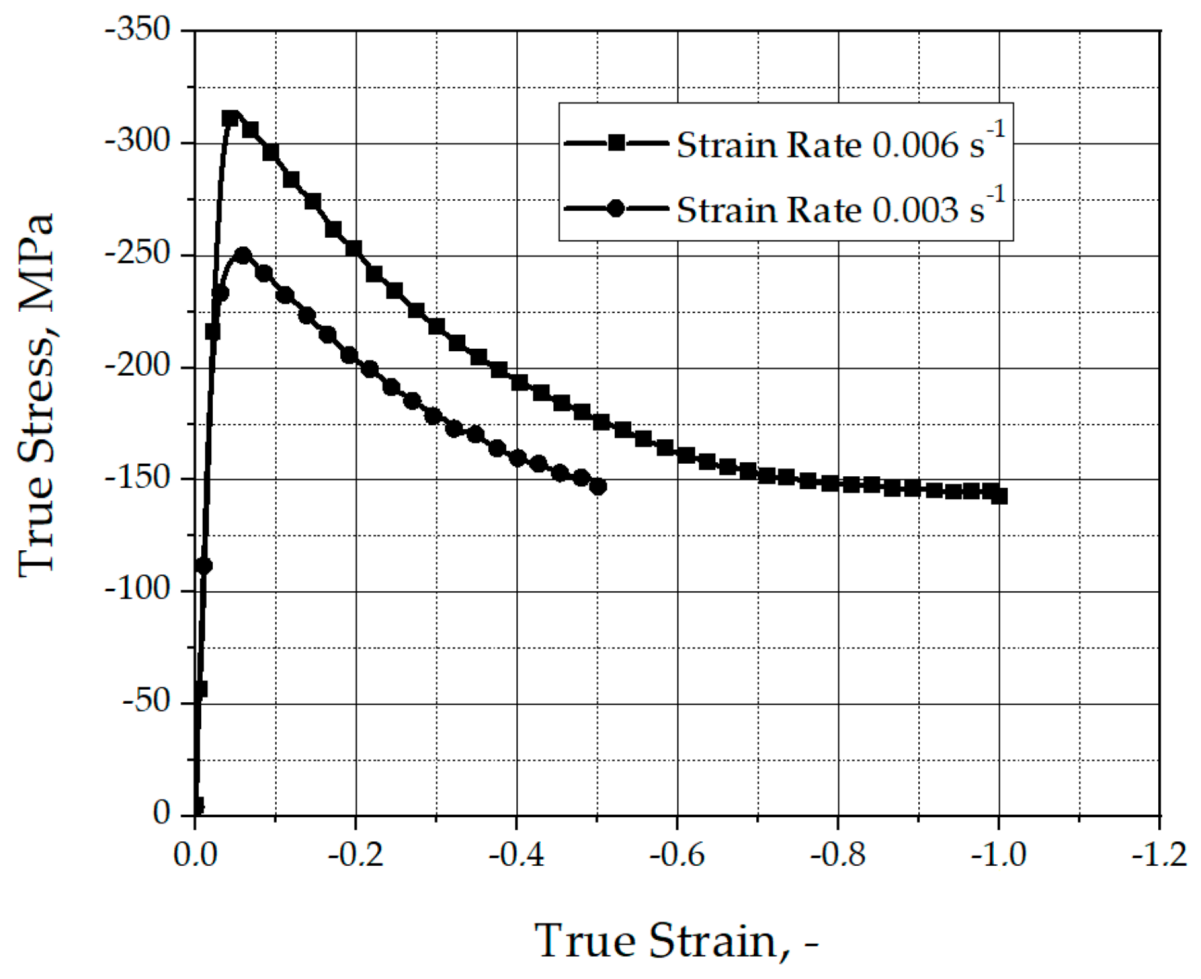
Plastic flow curves of sintered components produced in accordance with Variant III under conditions of continuous deformation at a temperature of 680 °C.

**Figure 9:**
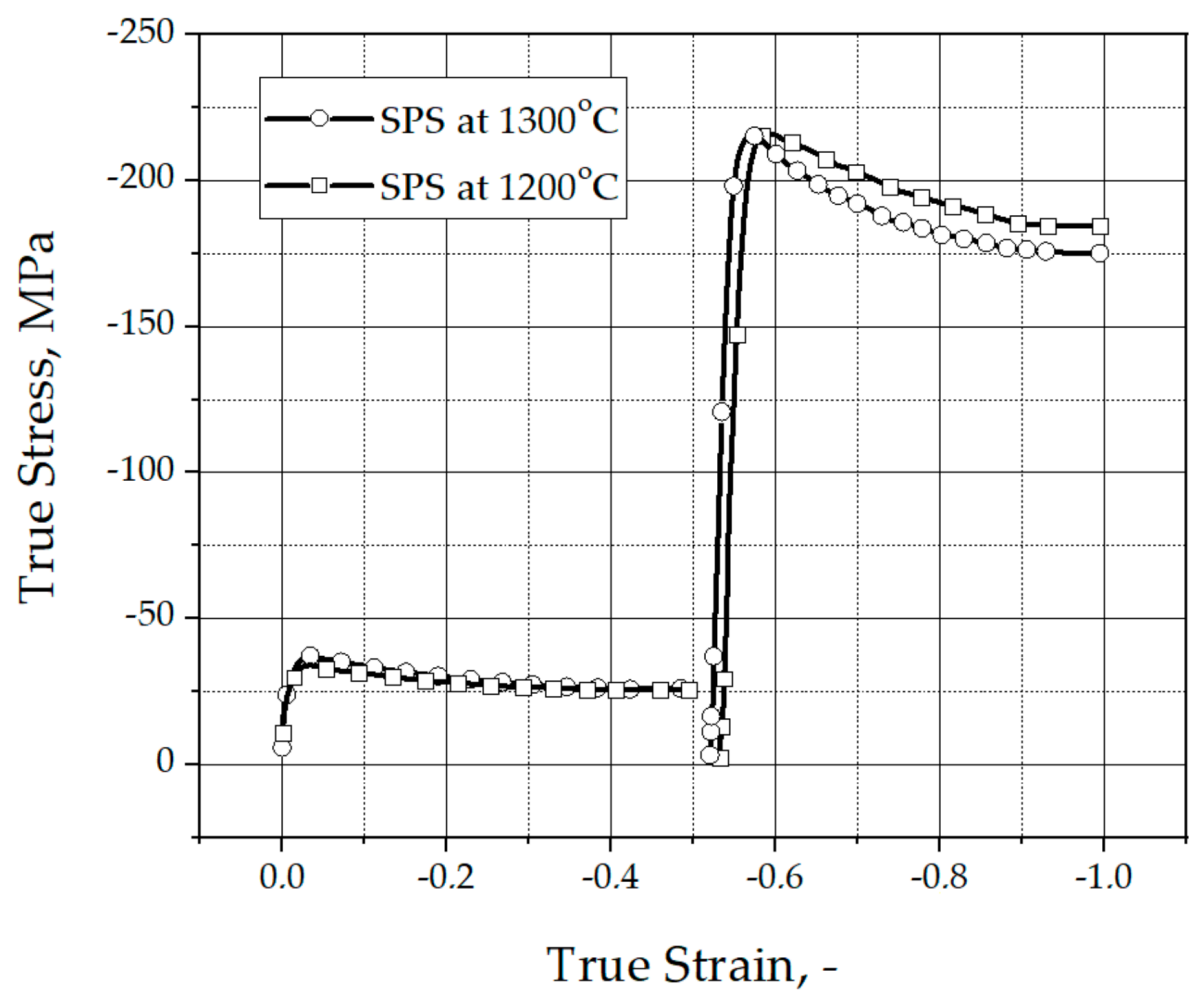
Plastic flow curves of sintered components produced in accordance with Variants I and III under the following deformation conditions: the first at 980 °C and the second at 680 °C.

Figure 8 shows the plastic flow curves during the compaction of the material obtained in the SPS process carried out according to Variant III, while Figure 9 shows the plastic flow curves during the two-stage compaction of the SPS parts according to Variants I and III.

**Figure 10:**
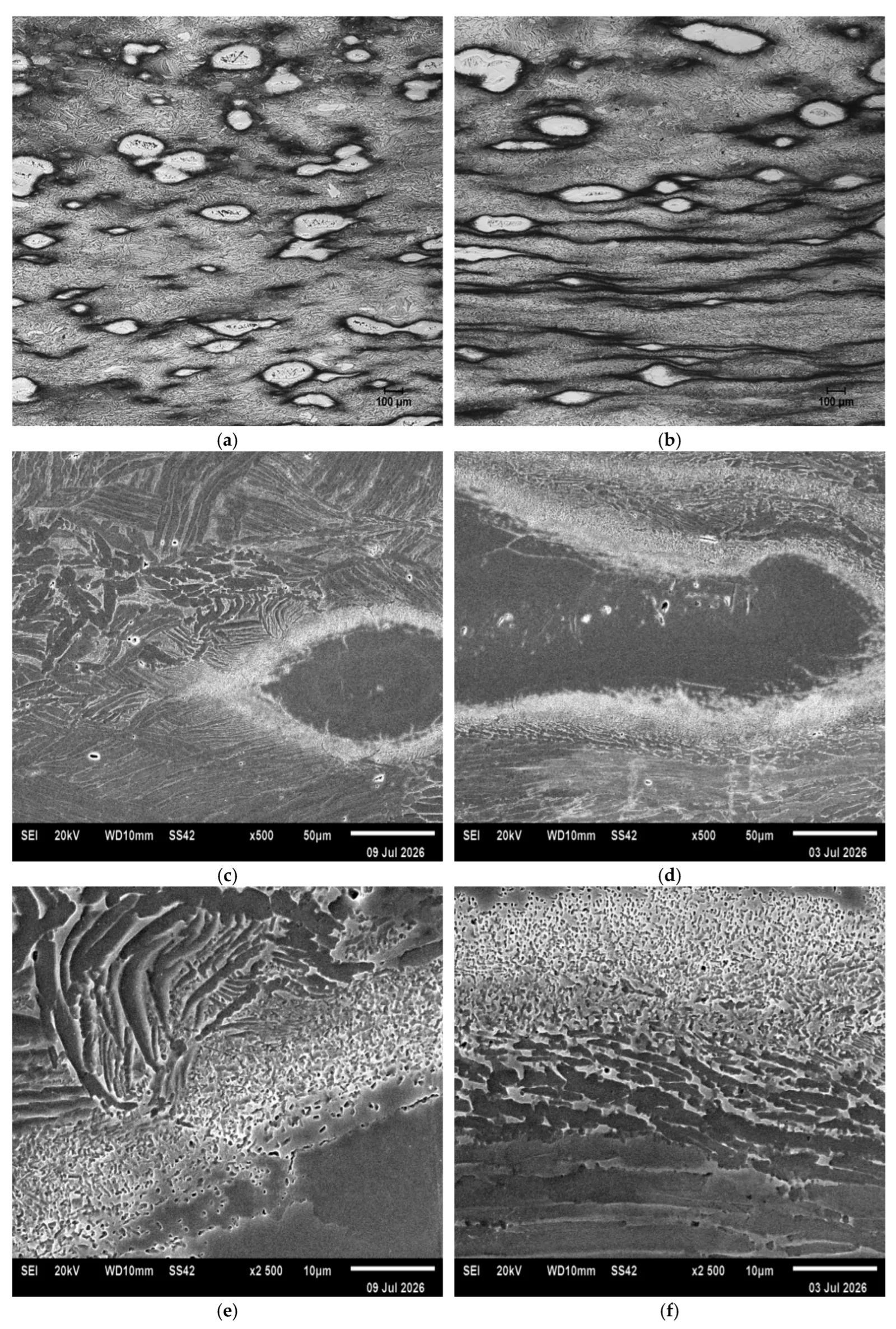
Microstructure of an SPS-produced casting according to Variant III (1300 °C, 10 min) that was deformed to a strain of ε = 0.5 (**a**,**c**,**e**) and ε = 1.0 (**b**,**d**,**f**); optical microscopy (**a**,**b**); SEM magnification 500× (**c**,**d**); SEM magnification 2500× (**e**,**f**).

The first stage of the research involved analysing the effect of continuous plastic deformation at 680 °C. Samples sintered by the SPS method for 10 min at 1300 °C (Variant III) were deformed to true strains of 0.5 and 1.0. An analysis of the plastic flow curves (Figure 8) reveals that during deformation, structural transformation processes occur in the material, associated with the recrystallisation of the β phase and a change in the morphology of the α phase, which, being harder, undergoes decomposition and partial spheroidization. The obtained data also indicate significant sensitivity of the material to strain rate. A twofold increase in strain rate led to more than a 20% increase in stress at the critical strain rate required to initiate dynamic recrystallisation. A low strain rate and a long deformation time allow the material to be deformed continuously without compromising its integrity. To explain and confirm the observed phenomena, Figure 10 presents examples of the material’s microstructures obtained using optical and scanning microscopy.

Figure 10 shows the microstructure images obtained for a material sintered using the SPS method Variant III and then deformed at 680 °C under continuous deformation of 0.5 at a rate of 0.003 s^−1^ (Figure 10a,c,e) and at a strain value of 1.0 with a strain rate of 0.006 s^−1^ (Figure 10b,d,f). The strain was applied at a temperature within the range where the two-phase structure occurs in the material. In both cases, the material underwent plastic deformation without loss of continuity, and macroscopic observations and those made with an optical microscope revealed no cracks. The structures obtained in both cases are highly heterogeneous but significantly different from those of the starting material. The smaller plastic deformation caused the elongation of regions heavily enriched with vanadium, which, due to the presence of the β phase, are characterised by high ductility and undergo recrystallisation or dynamic healing; furthermore, they exhibit lower hardness compared to the rest of the matrix. The transition zones, visible in Figure 10a as dark, elongated regions, were stretched in the direction of material flow. Globularization of the α phase was also observed in areas where the vanadium concentration corresponded to the chemical composition of the alloy under study. Areas depleted in vanadium and more heavily doped with aluminium—which stabilises the α phase—deformed in accordance with the direction of material flow, and the α-phase plates began to align along the lines of plastic flow. Within the regions with a significantly increased vanadium concentration, undissolved vanadium particles remain visible.

Increasing the strain to 1.0 and the strain rate to 0.006 s^−1^ resulted in the structure shown in Figure 10b,d,f. This structure exhibits distinct banding features associated with the initial segregation of alloying elements. Areas containing vanadium particle remnants underwent significant elongation and dynamic recrystallisation. Furthermore, the soft vanadium particle remnants were stretched in these areas (particularly in the central part of the specimen, where plastic strain accumulation occurred, as is clearly visible in Figure 10d). Under conditions of applied strain, the β phase undergoes intense dynamic recrystallization, and in the areas surrounding the bright β-phase fields—where the vanadium concentration decreases—an ultrafine-grained or even nanometric globular α-phase structure formed within the β-phase matrix (Figure 10f). In vanadium-depleted regions, the α-phase plates, which have been subdivided into smaller ones, align along the direction of the material’s plastic flow lines.

**Figure 11:**
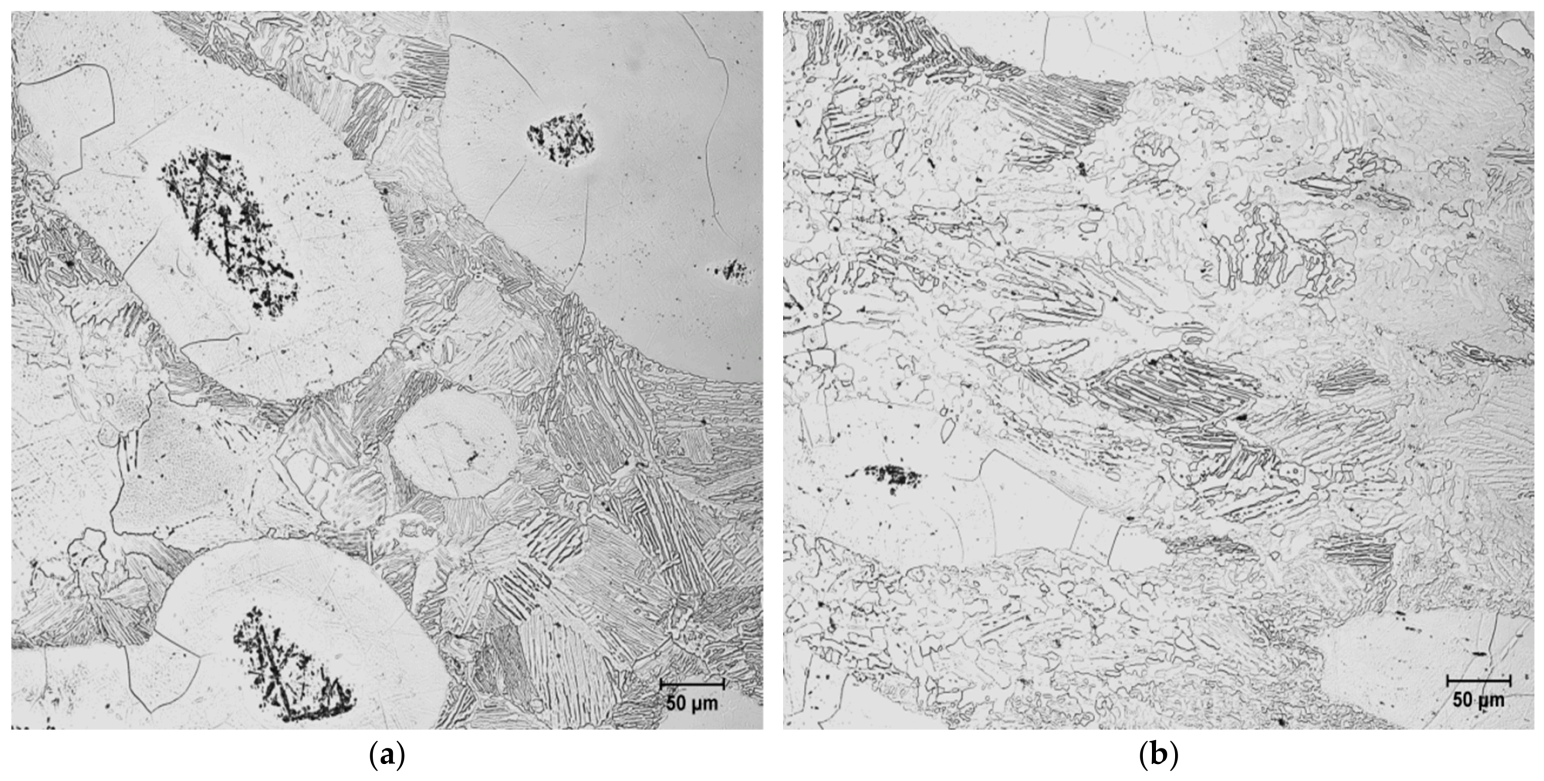
Microstructure of SPS-produced parts according to Variant III (1300 °C, 10 min) annealed at 920 °C for 2 h after plastic deformation of 0.5 (**a**) and 1.0 (**b**).

In the next stage of the study, the samples, after continuous deformation at 680 °C, were annealed at 920 °C in an argon atmosphere for 2 h and then cooled in air. Metallographic sections were prepared from the resulting material, and the microstructure images obtained from optical microscopy are shown in Figure 11.

Figure 11a shows the microstructure of a heat-treated specimen that had previously been plastically deformed at a strain rate of 0.003 s^−1^ with a strain of 0.5. Annealing was performed at 920 °C, within the β-phase stability range, to limit excessive grain growth and eliminate the highly dislocation-rich banded structure. As a result of this process, a microstructural transformation occurred, characterised by oval β-phase regions heavily enriched in vanadium and fine α-phase precipitates along grain boundaries. A typical two-phase structure formed around these regions, in which lamellar α-phase precipitates in the β-phase matrix are located within the region of former β-phase grains. Furthermore, remnants of undissolved vanadium particles are still visible within the bright titanium-vanadium regions. Annealing samples that had been deformed at a higher rate and to a greater degree resulted in the structure shown in Figure 11b. High plastic deformation caused significant elongation of vanadium-enriched areas and fragmentation of the remaining vanadium particles. During annealing, due to the high internal energy accumulated in the material, recrystallisation of the static β phase occurred more intensively, and vanadium diffusion mechanisms in titanium were reactivated. The result is a reduction in the areas heavily enriched with vanadium and the appearance of a greater amount of a two-phase structure in the material, consisting of plates of the α phase, precipitated during cooling, within the β-phase matrix. Furthermore, when comparing the two structures, it can be observed that after heat treatment of the more heavily deformed material (Figure 11b), the β-phase grains are smaller. This applies to both the primary grains, in which α-phase precipitates are present, and the grains in which no such precipitates were observed.

**Figure 12:**
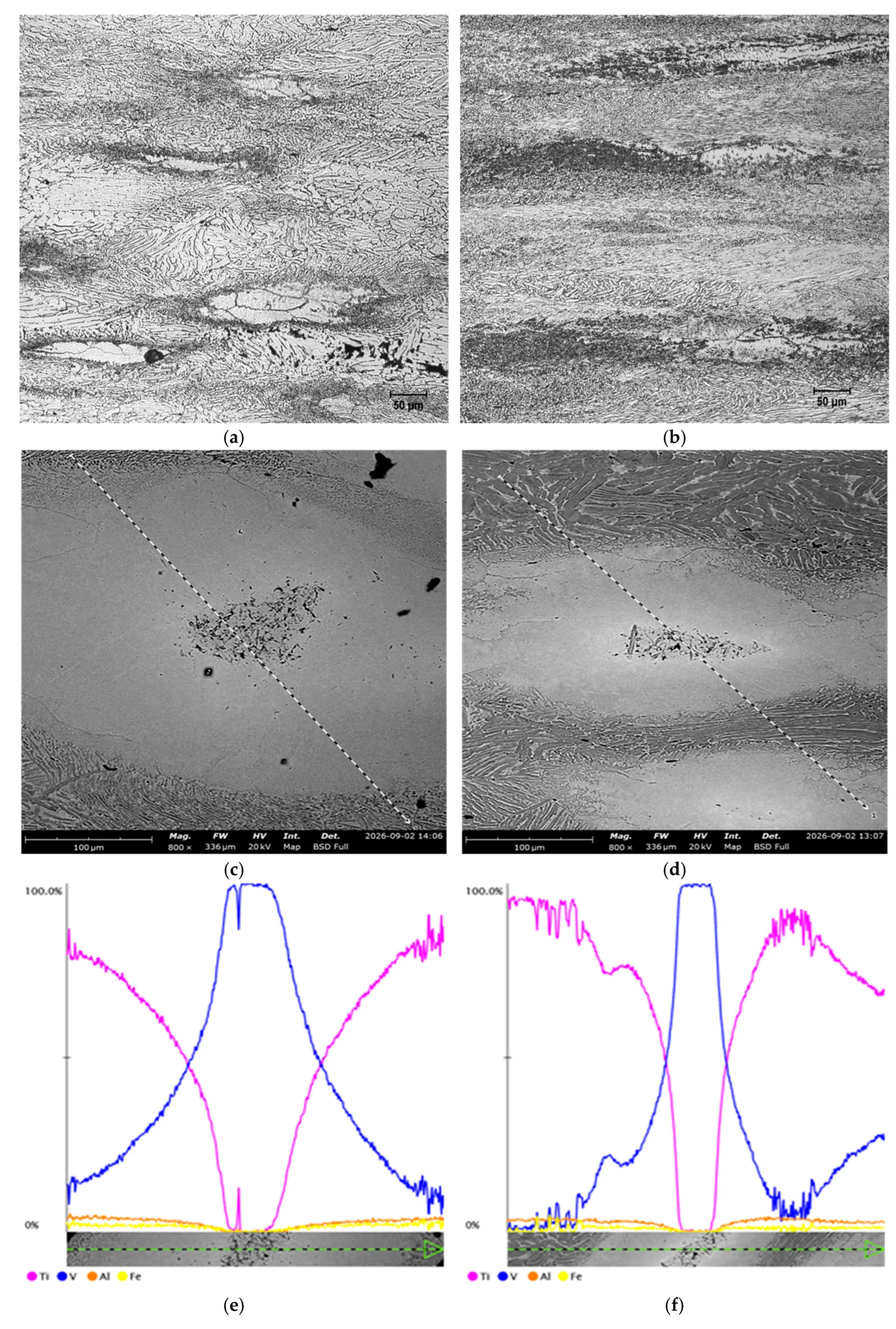
Microstructure of sintered specimens produced by the SPS method according to Variant I (1200 °C, 10 min) (**a**,**c**,**e**) and according to variant III (1300 °C, 10 min) (**b**,**d**,**f**) after thermomechanical treatment, along with a linear analysis of alloying element concentrations in areas with vanadium particle residues (**e**,**f**).

In the third stage of the study, an analysis was conducted on the material obtained by the SPS method according to Variant I (1200 °C, 10 min) and Variant III (1300 °C, 10 min), which was then subjected to thermomechanical treatment. The samples were deformed by applying two strains of 0.5 at a rate of 0.003 s^−1^. The first strain was applied at 980 °C, after which the sample was cooled at 2 °C/s to 680 °C, and the second strain was then applied. The resulting microstructures were observed using an optical microscope and scanning electron microscopy (SEM). The test results are presented in Figure 12.

Macroscopic observations of the samples after thermomechanical treatment confirmed that the material retained its continuity; therefore, it remains highly deformable even at 680 °C. Observations using optical microscopy (Figure 12a,b) revealed significant differences in the resulting microstructure, indicating a major influence of the material’s manufacturing history. Based on the microstructural analysis shown in Figure 12a, it can be observed that the bright regions (rich in vanadium) retained a relatively compact, though elongated, elliptical shape. Internal grain boundaries are visible within them, and furthermore, most particles have retained structural continuity (only isolated transverse cracks are visible). The matrix consists of distinctly thicker, better-developed, and elongated α-phase plates. The colonies of α-phase plates or lamellar arrangements are geometrically larger than in the material obtained from a sintered blank at 1300 °C, whose microstructure is shown in Figure 12b.

In the analysed specimen, under deformation, the light-coloured zones underwent severe compression and elongation into very thin bands, along with intense fragmentation. It is now difficult to find large, oval cores in the analysed area. This indicates that the initial transition zone around the particles, formed during pressing at 1300 °C, exhibited greater susceptibility to plastic deformation and was stretched during deformation at both 980 °C and 680 °C. At the same time, the matrix underwent significant refinement. Very dark, dense, and banded regions with an ultrafine-grained structure are visible, resulting from the refinement of colonies of α-phase platelets. During deformation at 680 °C, these areas underwent local fragmentation (dynamic globularization or mechanical fragmentation), thereby further refining the structure. Microscopic examination of the samples after thermomechanical treatment also revealed vanadium-enriched regions containing remnants of vanadium particles. These areas were located near the contact bases of the samples subjected to swaging with the tool and, therefore, underwent less deformation than the central part of the cylindrical samples, whose microstructure is shown in the optical microscopy images (Figure 12a,b). The microstructural observations were supplemented by a linear analysis of the distribution of elemental concentrations in selected areas containing undispersed vanadium particles (Figure 12c–f). The analysis confirmed the presence of undissolved vanadium-rich particles. The significant heterogeneity in the samples’ microstructures after compression indicates that higher levels of plastic deformation promote intense grain refinement, the morphology of which may indicate high strength and ductility in the material.

## 4. Summary

In the analysed specimen, under deformation, the light-coloured zones underwent severe compression and elongation into very thin bands, along with intense fragmentation. It is now difficult to find large, oval cores in the analysed area. This indicates that the initial transition zone around the particles, formed during pressing at 1300 °C, exhibited greater susceptibility to plastic deformation and was stretched during deformation at both 980 °C and 680 °C. At the same time, the matrix underwent significant refinement. Very dark, dense, and banded regions with an ultrafine-grained structure are visible, resulting from the refinement of colonies of α-phase platelets. During deformation at 680 °C, these areas underwent local fragmentation (dynamic globularization or mechanical fragmentation), thereby further refining the structure. Microscopic examination of the samples after thermomechanical treatment also revealed vanadium-enriched regions containing remnants of vanadium particles.

The research demonstrated that using the spark plasma sintering (SPS) method to produce the Ti-10V-2Fe-3Al alloy yields a material with a highly varied microstructure, the nature of which depends on the sintering process parameters and subsequent thermomechanical treatment. Despite achieving a high degree of material densification, the short duration of the SPS process did not ensure complete dissolution of the vanadium particles in the titanium matrix. As a result, the sintered structure contained vanadium-enriched regions, which also contained undissolved vanadium residues, as well as a matrix with a two-phase α + β structure.

Increasing the sintering temperature from 1200 °C to 1300 °C or extending the process time from 10 to 15 min affects the morphology of the microstructural components, particularly the grain size of the β phase and the size of the α phase plates. Microscopic observations, supplemented by chemical composition analysis, confirmed the presence of local variations in the concentration of alloying elements, which influence the course of phase transformations and the variation in the material’s mechanical properties. Microhardness measurements revealed significant differences among individual areas of the microstructure, with values ranging from 109 to 470 HV0.3. Such a wide variation indicates a significant influence of local chemical composition, phase type, and microstructural changes on the mechanical properties of the sintered material.

Studies of plastic deformation at 680 °C showed that the material can be deformed without loss of continuity, despite significant structural heterogeneity. It was demonstrated that, as the deformation value increased, a banded structure developed and dynamic recrystallisation processes intensified. These changes were accompanied by a transformation in the morphology of the α phase, including its refinement and globularization in selected areas of the material.

An additional factor—the heat treatment applied, which involved annealing the samples at 920 °C for 2 h after plastic deformation—led to further microstructural transformation. The development of static recrystallisation processes and the reactivation of vanadium diffusion in the titanium matrix were observed. This resulted in a reduction in the size of areas heavily enriched with vanadium, particularly in the material subjected to greater deformation, and an increase in the proportion of the α + β two-phase structure. It was also found that the material subjected to greater plastic deformation prior to annealing was characterised by smaller β-phase grains. Despite the observed changes, the material’s microstructure remained heterogeneous.

The most interesting effects of microstructural changes were caused by thermomechanical processing: deformation at the β-phase temperature, followed by cooling at 2 °C/s to 680 °C, and deformation in the deep two-phase region at a low rate. In both cases studied—Variant II (sintering temperature 1200 °C, time 10 min) and Variant III (sintering temperature 1300 °C, time 10 min)—a highly refined microstructure was obtained. It was also found that the higher sintering temperature, combined with the effect of large plastic deformation, enabled the fragmentation and stretching of vanadium-enriched regions—where the vanadium concentration exceeds 10%—in accordance with the material’s flow directions. The two-phase matrix, which precipitated during cooling from the pressing temperature (reheated to the β-phase temperature, deformed, cooled to 680 °C, and deformed again), underwent extremely strong refinement and globularization of the α-phase. This process enabled very fine-grained refinement of the α phase. A banded composite structure was obtained, consisting of bands of fine or very fine grains of vanadium-enriched β-titanium and bands with an ultrafine-grained two-phase microstructure. Such a material should exhibit unique mechanical properties, provide high strength while maintaining ductility. Given the presence of β-phase bands, it should also exhibit greater impact resistance. It should also be noted that a sufficient degree of deformation is required to produce such a structure, since in the same specimens, within the layers located near the interface between the material and the tool—which were not deformed sufficiently—a structure containing undissolved vanadium particles remained.

The test results indicate that, despite significant initial segregation in chemical composition, applying high plastic deformation enables the production of a heterogeneous functional material. In the future, the authors plan to conduct strength tests on material subjected to thermomechanical processing at higher deformation levels, using larger initial specimens.

## 5. Conclusions

1. The use of the spark plasma sintering (SPS) method allows for the production of a Ti-10V-2Fe-3Al alloy with a high degree of densification; however, it does not ensure complete homogenization of its chemical composition. Areas enriched in vanadium remain in the microstructure, including undissolved particles of this element, whose presence affects the phase structure and mechanical properties of the material.

2. Intensive thermomechanical treatment, involving deformation within the β-phase region, controlled cooling to 680 °C, and re-deformation in the α + β two-phase region, enables significant refinement and restructuring of the alloy’s microstructure. The development of a banded structure, globularization of the α phase, and the elongation and refinement of vanadium-enriched regions were observed. The development of such a microstructure depends on the magnitude of the deformation and its distribution throughout the material’s volume.

3. The resulting heterogeneous microstructure offers the potential to achieve a favourable combination of mechanical properties in the Ti-10V-2Fe-3Al alloy, particularly high strength while maintaining adequate ductility and increased resistance to impact loads. Verification of these potential benefits requires further strength and impact tests, as well as optimisation of the thermomechanical processing parameters.

## Figures and Tables

**Table 1:** Chemical composition of the alloy under study.

Material	Ti	V	Fe	Al
% by weight	85	10	2	3

**Table 2:** Sintering process parameters.

Variants	Temperature [°C]	Time [min]	Pressing Pressure [MPa]
Variant I	1200	10	50
Variant II	1200	15	50
Variant III	1300	10	50

**Table 3:** Results of density measurements for the sintered specimens.

Variants	Temperature [°C]	Time [min]	Density [g/cm^3^]
Variant I	1200	10	4.608
Variant II	1200	15	4.597
Variant III	1300	10	4.645

## Data Availability

The original contributions presented in this study are included in the article. Further inquiries can be directed to the corresponding author.
